# PLAC1 as a serum biomarker for breast cancer

**DOI:** 10.1371/journal.pone.0192106

**Published:** 2018-02-12

**Authors:** Hongyan Yuan, Vincent Chen, Marc Boisvert, Claudine Isaacs, Robert I. Glazer

**Affiliations:** 1 Department of Oncology and Lombardi Comprehensive Cancer Center, Georgetown University Medical Center, Washington, DC; 2 Washington Hospital Center, Washington, DC; University of South Alabama Mitchell Cancer Institute, UNITED STATES

## Abstract

Placental-specific protein 1 (PLAC1) is an X-linked trophoblast gene that is re-expressed in several malignancies, including breast cancer, and is therefore a potential biomarker to follow disease onset and progression. Sera from 117 preoperative/pretreatment breast cancer patients and 51 control subjects, including those with fibrocystic disease, were analyzed for the presence of PLAC1 protein as well as its expression by IHC in tumor biopsies in a subset of subjects. Serum PLAC1 levels exceeded the mean plus one standard deviation (mean+SD) of the level in control subjects in 67% of subjects with ductal carcinoma in situ (DCIS), 67% with HER2^+^ tumors, 73% with triple-negative cancer and 73% with ER^+^/PR^+^ tumors. Greater sensitivity was achieved using the mean+2 SD of control PLAC1 serum values, where the false positive rate was 3% and was exceeded by 38%, 40%, 60% and 43% of subjects with DCIS, HER2^+^, TNBC and ER^+^/PR^+^/HER2^-^ tumors. PLAC1 was detected in 97% of tumor biopsies, but did not correlate quantitatively with serum levels. There was no significant correlation of serum PLAC1 levels with race, age at diagnosis, body mass index (BMI) or the presence of metastatic disease. It remains to be determined whether PLAC1 serum levels can serve as a diagnostic biomarker for the presence or recurrence of disease post-surgery and/or therapy.

## Introduction

Breast cancer is a leading cause of cancer deaths among women and accounts for approximately 25% of all cancers [1]. It is a heterogeneous disease with varied morphologic and genetic alterations that poses a challenge to its diagnosis and treatment [2]. Currently, breast cancer diagnosis relies predominantly upon 2D and 3D mammography for the detection of invasive and *in situ* cancer [3], which have recall rates for further imaging in approximately 10% of subjects [4]. Attempts to improve the prognosis of breast cancer with serologic tests have not met with success so far [5], and biopsy using immunohistochemical (IHC) classification still remains the mainstay for diagnosing and categorizing this disease [6]. Given the recognized differences in breast cancer incidence, subtypes, and prognosis among women [7], it is important to evaluate potential biomarkers in the landscape of breast cancer subtypes ranging from DCIS to triple-negative breast cancer (TNBC) to determine if a simple blood test can enhance the diagnosis of this disease.

As a step toward this goal, we conducted a pilot study to determine whether serum levels of Placental-specific protein 1 (PLAC1) could distinguish between different subtypes and stages of breast cancer. PLAC1 is expressed at high levels on the surface of trophoblast cells in the placenta, at low levels in the testis, but is otherwise absent in normal somatic tissues [8]. PLAC1 is expressed in human fetal tissues [9], and circulating PLAC1 mRNA increases during pregnancy [10], and as a result of pre-eclampsia, fetal injury [11] and implantation failure [12]. Many genes normally expressed in the embryo become reactivated in cancer cells, and PLAC1 was the first such cancer/testis gene that related placentation to cancer [13]. Silva, et al. [8] first reported that PLAC1 RNA was expressed in human cancer cell lines covering 17 different malignancies, including breast cancer, and Koslowski [14] published similar findings [15]. Notably, circulating PLAC1 mRNA levels covering a 5-log range were detected in the majority of subjects with primary breast cancer, and were greater in women with estrogen receptor positive (ER^+^) tumors [14–16]. These findings were consistent with the prevalence of PLAC1 in luminal A and B breast cancer in comparison to TNBC [17], and that PLAC1 is among the top 1% of genes expressed 4-fold or more in subjects with invasive breast carcinoma [18]. In a similar context, PLAC1 expression detected by IHC was upregulated in other cancers, such as serous endometrial adenocarcinoma [9] and late stage colon and liver cancer [19–21]. Overall, these data suggest that PLAC1 may have diagnostic value as a tumor-selective biomarker in breast and other malignancies.

Since there are currently no serologic tests for breast cancer, we wished to determine whether circulating levels of PLAC1 protein could serve as a biomarker in preoperative and pretreatment breast cancer subjects ranging from DCIS to early and late stage hormone-dependent, HER2^+^ or triple-negative disease, and whether it correlated with its expression in tumor biopsies.

## Materials and methods

### Patient samples and study design

Study subjects were recruited through the Nontherapeutic Subject Registry (NTSR) Shared Resource, Lombardi Comprehensive Cancer Center (LCCC), Georgetown University Medical Center, and the Breast Cancer Surgery Clinic, MedStar Washington Hospital Center, through an Institutional Review Board approved protocol that allows for the sharing of deidentified data. Participants in the NTSR complete a core questionnaire revealing medical history, cancer diagnosis, and treatment history among other key data modules. Serum specimens and corresponding FFPE tissue blocks from cancer patients were obtained through a protocol approved by the Biospecimen Use Committee, Georgetown University Medical Center. Sera were collected from women with a diagnosis of breast cancer of the DCIS, HER2^+^, TNBC and ER^+^/PR^+^/HER2^-^ subtypes prior to surgery or treatment. Sera from women presenting for routine mammography or subjects with no known medical condition (“unremarkable”) served as a negative control group. Subjects with fibrocystic condition (FCD) were categorized separately. Diagnostic information for all subjects as well as ethnicity, race and body mass index (BMI) are listed in S1 Table, Supporting Information. Mammograms of control subjects, when available, were classified by the Breast Imaging Reporting and Data System (BIRADS) as 1 (negative). BMI was calculated from the formula, BMI = weight in kg/(height in cm)^2^.

Gene expression data for PLAC1 in several breast cancer cohorts were obtained from the Oncomine database, http://www.oncomine.org (S1 Fig, Supporting Information).

### PLAC1 assay

Serum PLAC1 protein levels were determined by ELISA assay (#MBS931690, MyBioSource). Sera were diluted 1:10 with saline, and concentrations were calculated by comparison to a standard curve using recombinant hPLAC1 (MyBioSource). Color development was stopped and absorbance measured at 450 nm using an automated plate reader. A positive reaction was defined as an absorbance value that exceeded the mean absorbance value of a comparably diluted negative control by one standard deviation.

### Immunohistochemistry

Tissue blocks were assessed for PLAC1 by IHC using antibody G-1 (sc-365919, Santa Cruz Biotechnology) at a dilution of 1:400, which was predetermined by titration with serial dilutions of antibody. Staining in the absence of primary antibody served as a negative control. Formalin-fixed, paraffin-embedded sections of tumor biopsies were prepared for H&E staining and IHC by the Tissue and Histopathology Shared Resource, LCCC. Antigen retrieval was carried out by incubation of tissue sections in 10 mM sodium citrate buffer (pH 6.0) for 20 min at a sub-boiling temperature in an electric steamer as previously described [22–24]. Endogenous peroxidase activity was quenched with 3% hydrogen peroxide for 10 min, and incubated for 30 min with blocking solution (10% goat serum in Tris-buffered saline), followed by incubation overnight at 4°C with the appropriate primary antibody diluted in blocking solution. Biotin-conjugated secondary antibodies were diluted in TBS containing 0.1% Tween-20 and incubated for 30 min at room temperature using the ABC Vectastain (Vector Laboratories) detection system and diaminobenzidine (Pierce), and slides were counterstained with Harris-modified hematoxylin (Thermo-Fisher, Inc.), dehydrated and mounted in Permount (Thermo-Fisher, Inc.). PLAC1 expression pattern criteria determined by IHC were scored as 0, when negative; 1 when expression was of low intensity, and present in isolated groups of PLAC1^+^ cells within a histological section, and 2 when expression was of high intensity and present in the majority of cells. Three high power fields (400X) were randomly chosen for counting PLAC1^+^ cells in each core of the slide. A total of ~1000 cells per core per patient and slides were reviewed by two well-trained researchers blinded to the patients’ clinical information. The TNM classification of breast cancer biopsies was determined by a board-certified surgeon and pathologist.

### Statistical analysis

Statistical significance of means±S.D. were evaluated using the two-tailed Student’s t test at *P*<0.05 using GraphPad Prism 7 software.

## Results

**Fig 1:**
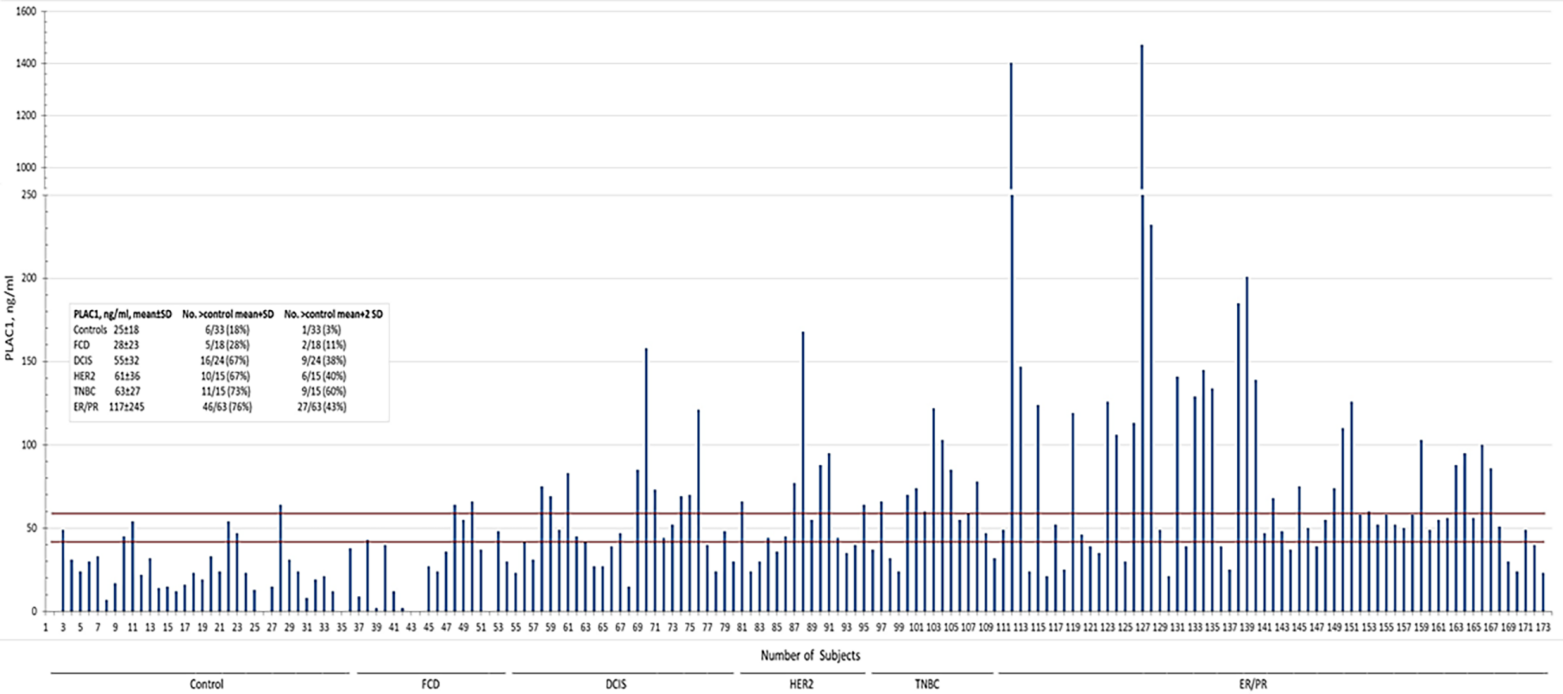
Serum PLAC1 levels in control and breast cancer subjects. Shown are serum levels from individual subjects. The mean plus one or two SD of PLAC1 serum levels in control subjects is indicated by the *lower* and *upper red* lines, respectively. The *inset* indicates the data for each subject category. *FCD*, fibrocystic disease.

Serum was obtained from control subjects, including those diagnosed with FCD, and patients diagnosed with DCIS, HER2^+^, TNBC and ER^+^/PR^-^/HER2^-^ breast cancer (Table 1). Analysis of serum in control subjects was 24±17 ng/ml, and did not differ significantly from subjects with FCD (Fig 1, Table 2). Among control subjects, 18% and 3% had levels that exceeded the mean+SD or mean+2 SD, respectively (Fig 1), and were considered to be the false-positive rates of the assay. PLAC1 levels in subjects with FCD did not differ significantly from control levels, but represented slightly higher percentages of 28% and 11% than control subjects based on the mean+SD and mean+2 SD respectively (Fig 1). Subjects with DCIS without microinvasion had a PLAC1 level of 57±32 ng/ml whereas, patients with HER2^+^, TNBC and ER^+^/PR^+^/HER2^-^ disease had PLAC1 levels of 61±36, 63±27 and 117±245 ng/ml, respectively (Fig 1, Table 2). Based on the mean+SD and mean+2 SD of control subjects, all cancer patients exceeded the percentages of control subjects by approximately four-fold and 12-20-fold, respectively (Fig 1, table inset).

**Table 1:** Characteristics of control and cancer patients. FCD, fibrocystic condition.

Characteristics	Controls	FCD	DCIS	HER2^+^	TNBC	ER^+^/PR^+^/HER2^-^
Age, years (range, mean)	19–84, 45	24–53, 42	32–84,51	35–73, 53	32–69, 48	31–79, 55
Number	33	18	24	15	15	63
Ethnic Origin, N (%)						
White	23 (67)	9 (50)	13 (54)	10 (67)	10 (67)	41 (65)
Black	6 (18)	8 (44)	7 (29)	4 (27)	4 (27)	18 (29)
Hispanic	0	0	0	0	0	0
Asian/Pacific Islander	3 (9)	1 (6)	1 (4)	0	0	2(5)
Other	1 (3)	0	3 (13)	1 (6)	1 (6)	2 (3)
BMI (range, mean)	18.9–33.7, 24.2	17.3–42.6, 29.0	18.7–48.9, 27.2	18.2.2–32.1, 26.3	19.4–32.0, 24.6	18.2–43.7, 28.1
Histology						
Ductal Carcinoma In Situ			24	0	0	0
Invasive Ductal Carcinoma			0	15	15	63
Invasive Lobular Carcinoma			0	0	0	0
Stage						
IA				8	6	36
IB				0	0	1
IIA				4	6	14
IIB				2	0	1
IIC				0	0	1
IIIA				0	2	7
IIIB				1	0	0
IIIC				0	1	3
Tumor Size:						
pTis (DCIS)			24	0	0	0
pT1mi (<1 mm), microinvasion			0	0	0	0
pT1a (>1 mm ≤ 5 mm)				6	6	10
pT1b (>5 mm ≤10 mm)						14
pT1c (> 10 mm ≤20 mm)						18
pT2 (>20 mm ≤ 50 mm)				3	4	18
pT3 (> 50 mm)				1	1	3
No. Involved Nodes:						
N0, no node metastasis				15	8	46
N1, metastases in 1–3 axillary nodes					2	8
N2, metastases in 4–9 axillary nodes						5
N3, metastases in ≥10 axillary nodes					3	4
Distant Metastasis:						
M0, no metastases			24	15	15	63

**Table 2:** Serum and biopsy PLAC1 in control and cancer subjects.

N	Subjects	Serum PLAC1, ng/ml	P-value	PLAC1 Biopsy Intensity (%)		Age	BMI	Race	
		(mean±SD)	vs. All Controls	Negative Low High	N			% White	% Black
33	Controls	24±17				45±18	24.2±4.1	70	18
18	FCD	28±23	NS			42±8	29.0±7.2	50	44
24	DCIS	57±32	<0.001	8 67 25	12	51±12	27.2±11.9	54	29
15	HER2^+^	61±36	<0.001	0 40 60	5	53±12	26.3±6.6	67	27
15	TNBC	63±27	<0.001	0 43 57	7	48±12	24.6±3.8	67	27
63	ER^+^/PR^+^/HER2^-^	117±245	<0.01	3 50 47	34	55±11	28.1±5.7	65	29

Shown are the mean±SD; P-values were determined by the two-sided Student's t test at a signficance level of 0.05 vs. Controls. NS, not significant (P>0.05). There were no significant differences between subject groups for age, BMI and race. N, number of subjects; FCD, fibrocystic condition.

**Fig 2:**
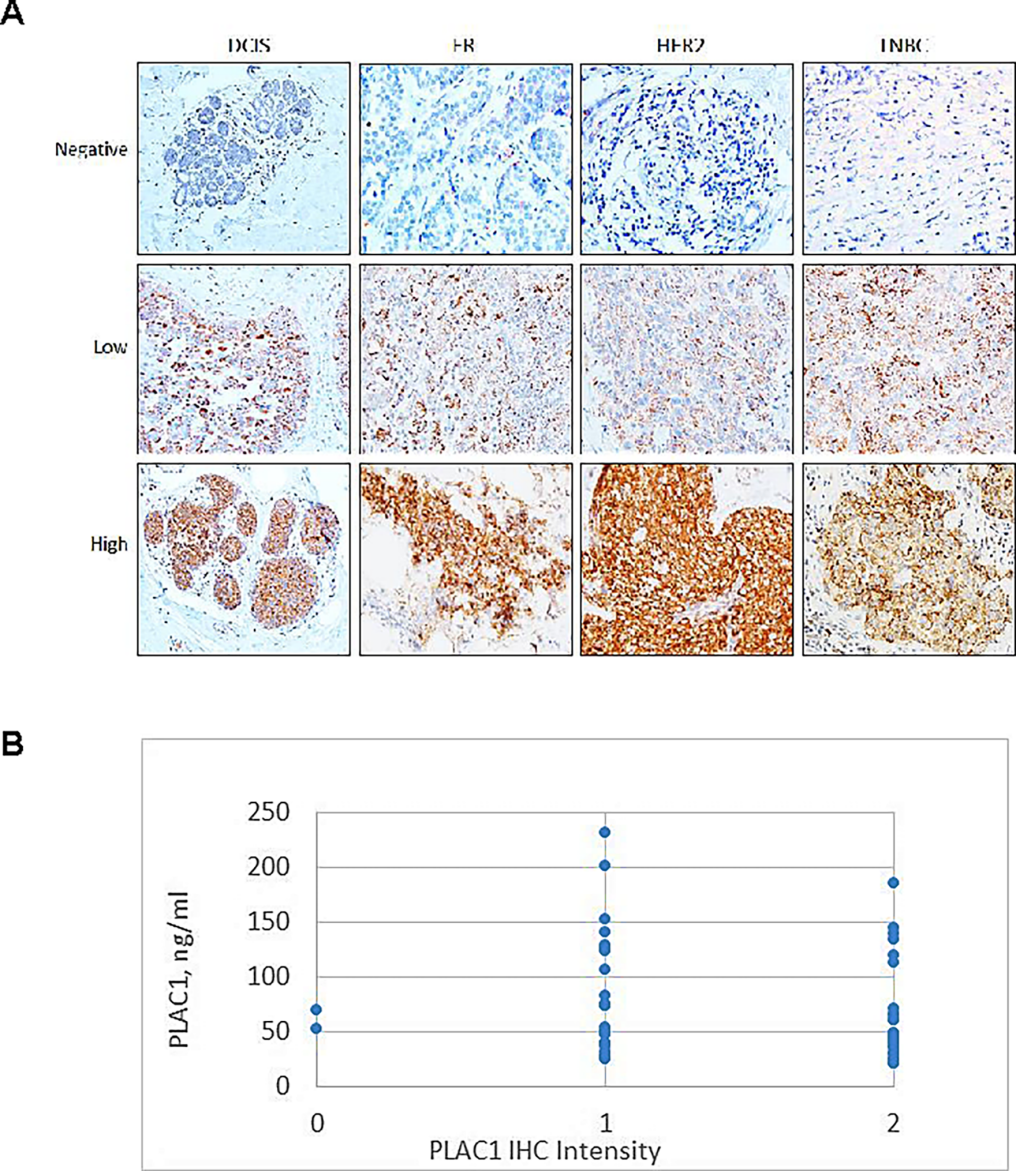
Immunochemical detection of PLAC1 in tumor biopsies and its correlation with serum PLAC1 levels. **A**, Examples of PLAC1 detection by IHC in tumor biopsies from subjects with DCIS, ER^+^/PR^+^/HER2^-^, HER2^+^ and TNBC. *Low* levels have less intensity and are more scattered than biopsies with *High* levels of PLAC1. **B**, Relationship between serum PLAC1 levels and PLAC1 expression in biopsies from the same patient based on the total number of subjects as indicated in Table 2. *0*, *1* and *2* represent negative, low and high staining intensity, respectively, as shown in **A**.

In 50% of the cases where tissue blocks from the same serum donor were available to determine PLAC1 expression by IHC, 92% of DCIS, 97% of ER^+^/PR^+^/HER2^-^ and 100% of HER2^+^ and TNBC cancers expressed PLAC1 (Table 2, Fig 2A). Although there did not appear to be a correlation between PLAC1 positivity in biopsies and serum PLAC1 levels (Fig 2B) or between serum PLAC1 levels and race, age or BMI (Table 2), the limited sample size would make this conclusion tentative.

## Discussion

PLAC1 protein expression in biopsies and circulating PLAC1 mRNA have been previously detected in subjects with breast cancer, particularly in those with hormone receptor-positive disease [14, 17, 25]. In the present study, we wished to determine if circulating levels of cell-free PLAC1 protein could serve as a diagnostic biomarker for breast cancer. Among the four breast cancer subtypes assessed, there was no correlation between serum PLAC1 levels, tumor histology, race, age, BMI, TNM staging or PLAC1 expression in tumor biopsies (Table 2). However, a statistically significant percentage ranging from 67% in DCIS to 73% in TNBC and ER^+^/PR^+^/HER2^-^ tumors expressed 2.4–4.9-fold higher levels of serum PLAC1 than control subjects. With false positive rates of 18% and 3% based on the mean+SD and mean+2 SD of control subjects, the net detection rate was 49% and 35% for DCIS, 49% and 37% for HER2^+^, 55% and 57% for TNBC and 55% and 40% for ER^+^/PR^+^/HER2^-^ cancers. These results are consistent with the relative fold changes in PLAC1 gene expression for DCIS and invasive ductal and lobular breast cancer in the Oncomine database and its relative absence in normal breast tissue (S1 Fig, Supporting Information). Whether the sensitivity of this assay is sufficient for its use as a diagnostic biomarker is uncertain. For example, since none of the DCIS subjects in our study exhibited microinvasion, it cannot be ascertained whether PLAC1 would be a useful biomarker to distinguish indolent disease from DCIS with microinvasion. Alternatively, high levels of serum PLAC1 may serve as a useful biomarker to follow response to chemotherapy and surgery, and subsequent drug resistance and recurrence. In this regard, we noted previously in MMTV-PPARd transgenic mice that PLAC1 RNA and protein were markedly upregulated at the onset and throughout tumor development, and were reduced following treatment with the mTOR inhibitor everolimus [24]. In estrogen-dependent breast cancer cells, PLAC1 expression was downregulated by antiestrogen treatment [15, 16] further suggesting that it may be a useful biomarker to follow treatment responsiveness and resistance in patients with hormone-dependent tumors.

In conclusion, the serum level of PLAC1 in breast cancer subjects with DCIS, HER2^+^, triple-negative and ER^+^/PR^+^ subtypes was a sensitive indicator of disease in 40–60 percent of subjects based on the mean+2 SD of levels in control subjects. The use of serum PLAC1 as biomarker in breast cancer merits further study.

## Supporting information

S1 Fig PLAC1 gene expression in cohorts of breast cancer subjects from the Oncomine database (http://www.oncomine.org). Show are waterfall plots of subjects vs. the log 2 scores for PLAC1 normalized to the median intensity in each cohort. Included are six different cohorts of breast cancer subjects of varying subtypes and one cohort of normal breast tissue showing a negative score.
(PDF)

S1 Table Subject characteristics and PLAC1 serum levels. PLAC1 serum levels (mean±SD) are given as ng/ml. FCD, fibrocystic disease; GERD, gastroesophageal reflux disease; IBS, irritable bowel syndrome; B, African-American; W, Caucasian; A, Asian/Pacific Islander; O, other, non-Hispanic.
(PDF)
