# Supplementary material for: PLAC1 as a serum biomarker for breast cancer
Source: PLoS One. 2018 Feb 12;13(2):e0192106. doi: 10.1371/journal.pone.0192106 (PMC5809008; doi:10.1371/journal.pone.0192106)
Supplement: S1 Fig — Show are waterfall plots of subjects vs. the log 2 scores for PLAC1 normalized to the median intensity in each cohort. Included are six different cohorts of breast cancer subjects of varying subtypes and one cohort of normal breast tissue showing a negative score. (PDF) [file pone.0192106.s001.pdf]

Farmer

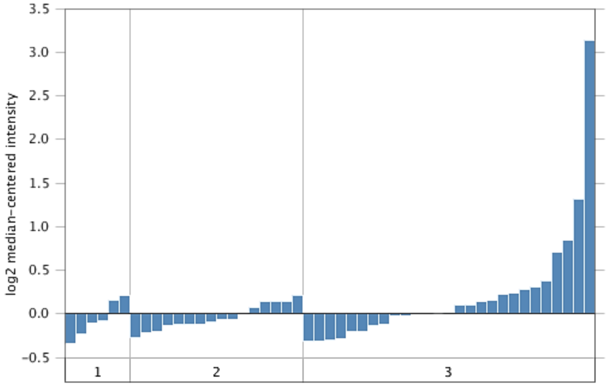

1. Apocrine Breast Carcinoma (6); 2. Basal-Like Subtype of Invasive Breast Carcinoma (16); 3. Luminal-Like Subtype of Invasive Breast Carcinoma (27)

Ma

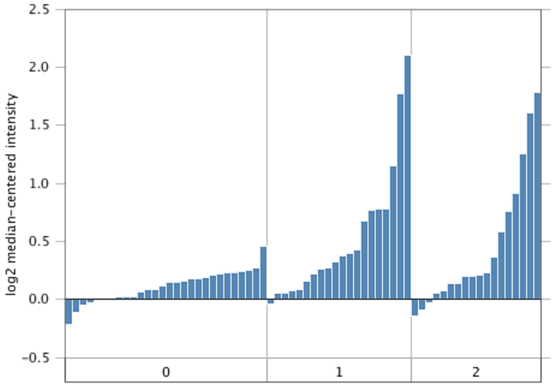

0. No value (28); 1. Ductal Breast Carcinoma in Situ (20); 2. Invasive Ductal Breast Carcinoma (18)

Bonnefoi

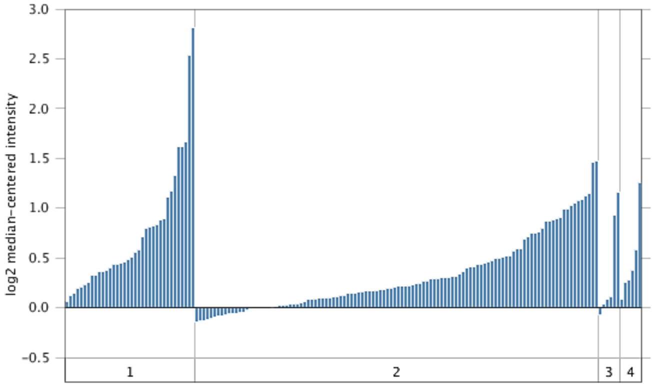

1. Breast Carcinoma (36); 2. Ductal Breast Carcinoma (112); 3. Invasive Breast Carcinoma (6); 4. Lobular Breast Carcinoma (6)

Lu

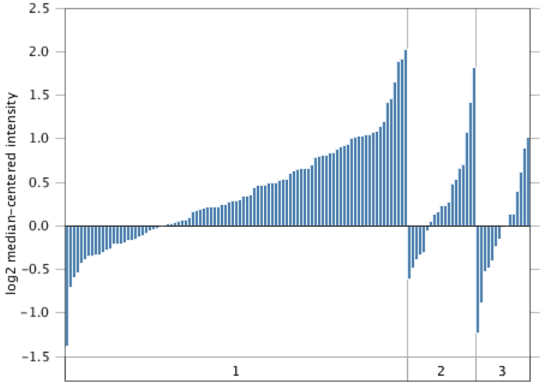

1. Ductal Breast Carcinoma (95); 2. Lobular Breast Carcinoma (19); 3. Mixed Lobular and Ductal Breast Carcinoma (15)

TCGA

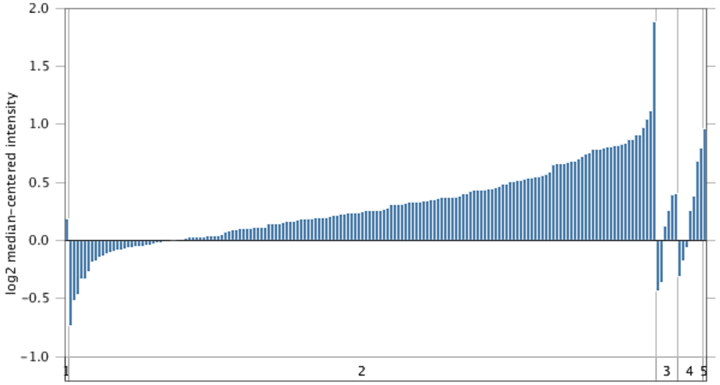

1. Invasive Breast Carcinoma (1); 2. Invasive Ductal Breast Carcinoma (163); 3. Invasive Ductal and Lobular Carcinoma (6); 4. Invasive Lobular Breast Carcinoma (7); 5. Mucinous Breast Carcinoma (1)

Loi

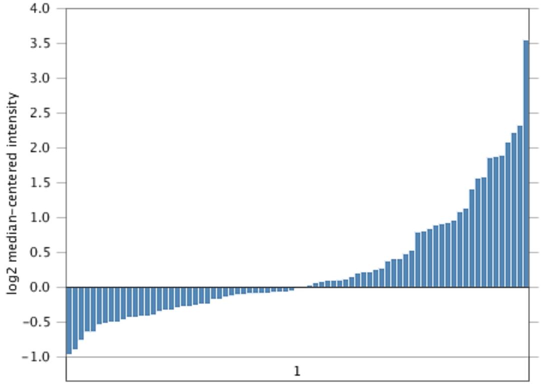

1. Invasive Breast Carcinoma (77)

TCGA

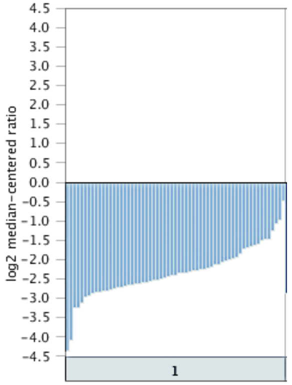

1. Breast (61)
