# Supplementary material for: PLAC1 as a serum biomarker for breast cancer
Source: PLoS One. 2018 Feb 12;13(2):e0192106. doi: 10.1371/journal.pone.0192106 (PMC5809008; doi:10.1371/journal.pone.0192106)
Supplement: S1 Table — PLAC1 serum levels (mean±SD) are given as ng/ml. FCD, fibrocystic disease; GERD, gastroesophageal reflux disease; IBS, irritable bowel syndrome; B, African-American; W, Caucasian; A, Asian/Pacific Islander; O, other, non-Hispanic. (PDF) [file pone.0192106.s002.pdf]

S1 Table. Subject Characteristics and PLAC1 Serum Levels. PLAC1 serum levels (mean±SD) are given as ng/ml. FCD, fibrocystic condition; GERD, gastroesophageal reflux disease; IBS, irritable bowel syndrome; B, African-American; W, Caucasian; A, Asian/Pacific Islander; O, other Non-Hispanic; NA, not available.

Subjects	Subject No.	Serum Level PLAC1, ng/ml	Diagnosis	Race	Age	BMI	Mammogram BIRADS Classification
**Controls**	32541	54	IBS	W	44	18.9	1
	33133	47	IBS	W	45	30.0	1
	34611	23	GERD	W	47	22.3	1
	34633	0	negative	O	45	21.8	1
	34636	13	ovarian cyst	W	73	24.5	1
	34657	0	IBS	W	58	24.5	1
	34756	0	negative	W	50	22.6	1
	35536	49	negative	B	84	26.5	1
	35934	15	endometriosis	B	44	25.4	NA
	35506	64	uterine bleeding, cervical radiculopathy	B	26	32.0	NA
	35858	24	depression	W	52	19.0	1
	35859	30	negative	W	54	23.9	1
	35834	31	negative	B	44	35.1	NA
	35868	33	negative	W	69	20.9	NA
	35871	7	negative	W	63	22.2	1
	35876	17	negative	W	70	26.7	1
	35879	45	negative	B	23	25.7	NA
	35880	54	negative	A	19	22.3	NA
	35881	22	negative	W	27	24.2	NA
	35891	32	negative	W	24	22.0	NA
	35893	8	asthma	A	25	33.7	NA
	35895	19	arthritis	W	57	22.9	1
	35896	14	unremarkable	W	66	26.7	1
	35899	21	hypertension	W	69	17.3	1
	35906	15	unremarkable	W	69	26.9	1
	35912	12	negative	W	46	21.9	NA
	35924	0	pituitary adenoma	W	24	28.3	1
	35912	12	negative	W	46	21.9	NA
	35933	16	negative	W	26	20.9	NA
	35962	23	negative	B	29	19.9	NA
	35963	19	negative	A	29	22.3	NA
	35967	33	negative	W	27	23.4	NA
	36037	24	negative	W	23	21.5	NA
**FCD**	32523	9	FCD	B	42	29.3	
	32525	43	FCD	B	29	21.5	
	33114	2	FCD	B	47	25.3	
	33115	40	FCD	W	24	26.3	
	33125	12	FCD	W	41	22.1	
	33131	2	FCD	A	31	26.4	
	33415	0	FCD	B	44	33.6	
	34628	0	FCD	B	53	38.6	
	34713	27	FCD	W	34	17.3	
	34754	24	FCD	W	43	23.4	
	34757	36	FCD	W	34	26.8	
	35554	64	FCD	B	43	42.6	
	35627	55	FCD	B	53	38.6	
	35797	66	FCD	B	52	39.1	
	35872	37	FCD	W	47	23.1	
	35888	0	FCD	W	46	35.1	
	35897	48	FCD	W	48	27.4	
	36028	30	FCD	B	44	25.4	
**DCIS**	31996	31	Stage 0: pTis	O	48	24.3	
	32005	75	Stage 0: pTis	O	32	28.3	
	32018	69	Stage 0: pTis	B	43	26.7	
	32032	49	Stage 0: pTis	W	45	19.6	
	32161	83	Stage 0: pTis	W	50	23.0	
	32165	45	Stage 0: pTis	W	52	22.3	
	32421	42	Stage 0: pTis	B	47	31.1	
	32822	27	Stage 0: pTis	O	45	22.9	
	33533	27	Stage 0: pTis	W	84	22.6	
	33843	39	Stage 0: pTis	W	51	20.9	
	34578	47	Stage 0: pTis	W	37	25.9	
	34629	15	Stage 0:pTis	W	46	48.9	
	35513	85	Stage 0: pTis	B	70	37.1	
	35556	158	Stage 0: pTis	B	57	27.3	
	35559	73	Stage 0: pTis	W	48	18.7	
	35655	44	Stage 0: pTis	B	45	40.0	
	35678	52	Stage 0: pTis	B	62	28.5	
	35843	69	Stage 0: pTis	A	59	23.7	
	35926	70	Stage 0: pTis	W	74	25.7	
	35941	121	Stage 0: pTis	W	52	30.0	
	35956	40	Stage 0: pTis	B	42	28.9	
	35959	24	Stage 0: pTis	W	40	29.4	
	36042	48	Stage 0: pTis	W	44	26.6	
	36056	30	Stage 0: pTis	W	51	19.5	

**HER2+**	32137	66	Stage 2B: pT2 N0 M0; IDC	W	35	22.6
	32195	24	Stage 1A: pT1 N0 M0; IDC	W	54	25.7
	32210	30	Stage 2A: pT2 N0 M0; IDC	W	35	18.2
	33997	44	Stage 3B: pT1 N1 M0; IDC	B	73	28.1
	34156	36	Stage 2A: pT2 N0 M0; IDC	O	32	23.5
	35618	45	Stage 1A: pT1c N2 M0; IDC	B	68	31.6
	35630	77	Stage 1A: pT1a N0 M0; IDC	W	41	28.8
	35653	168	Stage 1A: pT1 N0 M0; IDC	W	67	24.9
	35663	55	Stage 1A: pT1c N0 M0; IDC	W	67	30.9
	35674	88	Stage 1A: pT1a N0 M0; IDC	W	55	32.1
	35684	95	Stage 2A: pT1c N1 M0; IDC	W	60	24.2
	35875	44	Stage 2A: pT2 N0 M0; IDC	W	49	26.4
	35935	35	Stage 1A: pT1c N0 M0; IDC	W	43	24.7
	35977	40	Stage 2B: pT2 N1 M0; IDC	B	57	22.7
	36113	64	Stage 1A: pT1c N0 M0; IDC	B	63	30.3
**TNBC**	32089	37	Stage 1A: pT1a N0 M0; IDC	W	69	19.4
	32127	66	Stage 2A: pT2,N0 M0; IDC	W	45	25.6
	32137	32	Stage 2A: pT2b N0 M0, IDC	W	36	22.6
	32195	24	Stage 1A: pT1a N0 Mo, IDC	W	55	25.7
	32205	70	Stage 1A: pT1a N0 M0; IDC	W	60	21.0
	33463	74	Stage 3A: pT2 N2 M0; IDC	B	49	23.1
	34500	60	Stage 2A: pT2 N0 M0; IDC	W	35	24.0
	35482	122	Stage 1A: pT1c N0 M0; IDC	O	41	20.4
	35525	103	Stage 2A: pT1c N1 M0; IDC	W	51	21.5
	35639	85	Stage 1A: pT1b N0 M0; IDC	B	64	32.0
	35708	55	Stage 2A: pT2 N0 M0; IDC	W	62	27.6
	35842	59	Stage 2A: pT2 N0 M0; IDC	W	29	31.7
	35864	78	Stage 1A: pT1b N0 M0; IDC	B	54	26.6
	35990	47	Stage 3A: pT2 N1 M0; IDC	W	43	25.4
	36196	32	Stage 3C: pT2 N3 M0; IDC	B	32	22.9
**ER+/PR+/HER2-**	31861	49	Stage 1A: pT1b N0 M0; IDC	W	49	25.3
	31869	1402	Stage 1A: pT1c N0 M0; IDC	B	61	34.0
	31874	147	Stage 2A: pT2 N0 M0; IDC	B	53	36.8
	31929	24	Stage 2A: pT2 N0 M0; IDC	B	32	28.0
	31930	124	Stage 2A: pT2 N0 M0; IDC	W	68	36.9
	31948	21	Stage 2A: pT3 N2 M0; IDC	W	75	22.3
	31950	52	Stage 3C: pT2 N3 M0; IDC	W	52	29.7
	31994	25	Stage 1A: pT1c N0 M0; IDC	O	50	29.5
	32013	119	Stage 3C: pT2 N3 M0; IDC	W	41	31.1
	32090	46	Stage 1A: pT1c N0 M0; IDC	W	47	37.6
	32095	39	Stage 2A: pT2 N0 M0; IDC	W	66	25.0
	32128	35	Stage 1A: pT1c N0 M0; IDC	W	46	35.3
	32143	126	Stage 1A: pT1c N0 M0; IDC	W	45	27.9
	32144	106	Stage 1A: pT1b N0 M0; IDC	B	50	28.1
	32177	30	Stage 1A: pT1c N0 M0; IDC	W	52	18.2
	32179	113	Stage 1A: pT1c N0 M0; IDC	W	41	24.3
	32191	1472	Stage 1A: pT1b N0 M0; IDC	W	53	23.0
	32204	232	Stage 1A: pT1a N0 M0 IDC	W	57	24.9
	32226	49	Stage 1A: pT1c N0 M0; IDC	W	64	29.6
	32236	21	Stage 1A: pT1b N0 M0; IDC	B	49	29.9
	32277	141	Stage 1A: pT1c N0 M0; IDC	W	79	28.1
	32282	39	Stage 2A: pT2 N0 M0; IDC	O	69	24.1
	32511	129	Stage 2A: pT2 N0 M0; IDC	W	37	40.5
	32517	145	Stage 1A: pT1a N0 M0; IDC	W	60	33.8
	33021	134	Stage 2C: pT1c N3 M0; IDC	B	37	40.5
	33113	39	Stage 1B: pT1c N1 M0; IDC	W	50	30.7
	33132	25	Stage 1A: pT1b N0 M0; IDC	B	44	23.1
	33419	185	Stage 1A: pT1a N0 M0; IDC	W	55	24.0
	33492	201	Stage 2A: pT2 N2 M0; IDC	W	49	29.4
	33550	139	Stage 2A: pT2 N0 M0; IDC	B	49	43.7
	33757	47	Stage 1A: pT1c N0 M0; IDC	W	52	25.9
	33808	68	Stage 1A: pT1b N0 M0; IDC	W	48	19.2
	33821	48	Stage 3A: pT2 N2 M0; IDC	W	60	23.9
	33908	37	Stage 2B: pT2 N1 M0; IDC	B	50	26.7
	33916	75	Stage 2A: pT2 N1 M0; IDC	W	35	34.0
	34112	50	Stage 1A: pT1b N0 M0; IDC	W	56	19.6
	34123	39	Stage 2A: pT2 N1 M0; IDC	W	56	30.7
	34282	55	Stage 1A: pT1b N0 M0; IDC	B	61	31.5
	34293	74	Stage 1A: pT1b N0 M0; IDC	W	75	19.7
	34589	110	Stage 1A: pT1b N0 M0; IDC	A	51	23.3
	35442	126	Stage 3A: pT3 N1 M0; IDC	W	57	24.3
	35464	58	Stage 1A: pT1a N0 M0; IDC	B	64	38.2
	35467	60	Stage 1A: pT1c N0 M0; IDC	W	64	27.4
	35469	52	Stage 3C: pT1b N3 M0; IDC	B	65	35.7
	35522	58	Stage 3A: pT2 N2 M0; IDC	B	51	20.7
	35524	52	Stage 3A: pT2 N0 M0; IDC	B	73	26.2
	35566	50	Stage 1A: pT1a N0 M0; IDC	W	54	26.2
	35602	58	Stage 1A: pT1b N0 M0; IDC	W	44	24.6
	35629	103	Stage 1A: pT1c N0 M0; IDC	W	51	23.0

	35640	49	Stage 1A: pT1b N0 M0; IDC	B	73	24.3
	35663	55	Stage 1A: pT1c N0 M0; IDC	W	67	30.9
	35668	56	Stage 1A: pT1b N0 M0; IDC	W	48	20.9
	35674	88	Stage 1A: pT1a N0 M0; IDC	W	55	32.1
	35684	95	Stage 2A: pT1c N1 M0; IDC	W	60	24.2
	35688	56	Stage 1A: pT1c N0 M0; IDC	A	59	26.6
	35706	100	Stage 1A: pT1a N0 M0; IDC	B	69	32.2
	35746	86	Stage 1A: pT1a N0 M0; IDC	W	51	25.1
	35778	51	Stage 1A: pT1a N0 M0; IDC	W	54	24.9
	35791	30	Stage 2A: pT1a N1 M0; IDC	W	31	26.5
	35806	24	Stage 1A: pT1c N0 M0; IDC	B	56	32.4
	35850	49	Stage 2A: pT2 N0 M0; IDC	W	60	26.1
	35981	40	Stage 3A: pT3 N1 M0; IDC	W	76	21.4
	36158	23	Stage 3A: pT2 N2 M0; IDC	B	45	25.2
